# Runoff and soil loss in biocrusts and physical crusts from the Tabernas Desert (southeast Spain) according to rainfall intensity

**DOI:** 10.3389/fmicb.2023.1171096

**Published:** 2023-05-24

**Authors:** Roberto Lázaro, Cayetana Gascón, Consuelo Rubio

**Affiliations:** ^1^Estación Experimental de Zonas Áridas (CSIC), Almería, Spain; ^2^SGS Tecnos SAU, Madrid, Spain

**Keywords:** biocrusts, soil crust dynamics, semi-arid, rainfall simulation, raindrop impact, splash erosion

## Abstract

Biological soil crusts (biocrusts) influence hydrological and erosive processes in drylands, and their effects increase with hypothetic successional development. Runoff and raindrops, both dependent on rain intensity, are among the main causes of erosion in these areas. However, little is known about the existence of soil loss nonlinearity in relation to rain intensity and crust types; this nonlinearity could control biocrust succession and dynamics. The assumption of biocrust types as successional stages, which allow space-for-time sampling, makes it advisable to include all the successional stages when exploring possible nonlinearity. We considered seven types of crusts, three physical and four biological. We created four rainfall intensity levels in controlled laboratory conditions: 18, 60, 120, and 240 mm/h. In all but the last, we conducted the experiments at two levels of antecedent soil moisture. Generalized Lineal Models enabled us to test for differences. These analyses confirmed previous knowledge regarding the significant effect of rainfall intensity, crust type and antecedent soil moisture and their interactions on runoff and soil loss, despite the small sample size of the sample units. For example, runoff, and particularly soil loss, decreased along succession. Moreover, some results were novel: the runoff coefficient increased only up to 120 mm/h of rain intensity. A decoupling between runoff and soil loss occurred at high intensities. Soil loss increased as rainfall intensity increased only up to 60 mm/h, and then it decreased, mainly due to physical crusts, because of the formation of a water sheet on the surface due to the incoming rainwater exceeding the drainage capacity. Although soil loss was greater in the incipient cyanobacteria than in the most developed lichen biocrust (Lepraria community), the protection provided by any biocrust against soil loss was great compared to the physical crust, and almost as strong at all rain intensities. Soil loss increased with antecedent soil moisture only in physical crusts. Biocrusts resisted the rain splash even at a rainfall intensity of 240 mm/h.

## 1. Introduction

Bare soil usually forms physical crusts on its surface due to raindrop impacts. Biological soil crusts (biocrusts) are communities composed mainly of microorganisms (bacteria), algae, fungi, lichens and mosses developing at the soil surface. They show high species richness, but they may be dominated by cyanobacteria, lichens or mosses, thus resulting in different biocrust types. Because some biocrust components have a greater colonizing capacity than others and are frequently replaced by others over time, it is often considered that biocrust types can represent different stages of succession (Belnap and Eldridge, 2003; Belnap et al., 2013; Drahorad et al., 2013; Weber et al., 2016). However, such succession does not necessarily occur at every site (Kidron, 2019; Lázaro et al., 2021), because the types are often associated with microhabitats and segregated in space, so in the Tabernas Desert (Cantón et al., 2004; Pintado et al., 2005, 2010) as at North America (Belnap, 2006; Bowker and Belnap, 2008) and at the Negev desert (Israel; Veste et al., 2001; Veste and Littmann, 2006). In the Tabernas Desert, our study area, only some biocrusts (i.e., cyanobacterial) can colonize any habitat. The lichenic biocrust characterized by *Lepraria* is exclusive to the shadiest slopes, whereas the other lichen biocrusts are intermediate. Thus, although lichens, accompanied finally by mosses, progressively replace cyanobacteria (Lázaro et al., 2008), this happens at different speeds depending on the features of the microhabitat. In fact, such replacement hardly occurs in the sunniest places, where cyanobacterial biocrust is practically permanent at the human life scale, according to our direct observations over the last 35 years. Despite this, replacement over time is frequent enough in space for many authors to associate biocrust types with successional development (see Dümig et al., 2014; Gypser et al., 2016).

Biocrusts have multiple and important ecosystem functions, particularly in drylands. They increase the availability of nitrogen (Castillo-Monroy et al., 2010), affect carbon fluxes (Cantón et al., 2014; Ladrón de Guevara et al., 2014), trap seeds (Escudero et al., 2007) and modify soil moisture (Chamizo et al., 2013) and temperature (Lázaro et al., 2008). Soil carbon content correlates with cryptogamic biomass through biocrust successional stages (Gypser et al., 2015). Biocrusts affect infiltration and erosion by constituting a physical barrier (Lázaro et al., 2021) and by making the soil rougher (Rodríguez-Caballero et al., 2012). This influences runoff behavior (Rodríguez-Caballero et al., 2013), initiates interactions between soil-surface components (Rodríguez-Caballero et al., 2014) and favors the patchy vegetation pattern that is typical of semi-arid areas (Mayor et al., 2009; Chamizo et al., 2016). Biocrusts can control the landscape vegetation pattern by limiting infiltration thus counteracting the rainfall gradient (Veste, 2011). The biocrust’s effect of decreasing water and wind erosion is, possibly, its most important function and can have geomorphological consequences at the landscape scale (Lázaro et al., 2022). Biocrusts decrease erosion through several mechanisms, such as forming a physical barrier, retaining sediments by increasing surface roughness, and increasing aggregate stability (Eldridge and Kinnell, 1997). This effect has been reported in Australia (Eldridge and Greene, 1994; Eldridge and Leys, 2003), the United States (Bowker et al., 2008; Belnap et al., 2013), China (Bu et al., 2015; Gao et al., 2019), Israel (Kidron and Yair, 1997; Kidron et al., 1999) and Niger (Malam Issa et al., 2011). Previous research in our study area (the Tabernas Desert, Almería, Spain), based on *in situ* rainfall simulations or in runoff plots under natural rainfall, showed that biocrusts protected the soil surface (Calvo-Cases et al., 1991; Solé-Benet et al., 1997; Chamizo et al., 2012b, 2017) depending on biocrust type (Chamizo et al., 2012a, 2015; Lázaro and Mora, 2014) and coverage (Chamizo et al., 2010).

In semi-arid zones, the main mechanism generating runoff is infiltration excess (Horton, 1933), which depends on the intensity and amount of rainfall. In turn, water erosion depends on raindrops (that is, also on the intensity and amount of rainfall) and on runoff. Simulating rain *in situ* provides valuable data but entails considerable logistical effort, and often a single rain intensity is used. In addition, it is very difficult to perform *in situ* simulations on biocrust types with a large slope angle; so, the biocrust characterized by Lepraria at Tabernas Desert remains largely unknown (but see Lázaro et al., 2021). Thus, although much information about runoff and soil loss in biocrusts is available, little is known about the possible thresholds (as Lázaro and Mora, 2014 suggested) or nonlinearity in those processes due to rain intensity. These possible thresholds would provide valuable information about biocrust dynamics, which could be crucial when considering biocrusts’ importance in semi-arid ecosystems due to the scarcity of vascular vegetation.

The purpose of this study was to evaluate the erodibility of the crusts present in the Tabernas Desert at a wide range of rainfall intensities using laboratory rain simulations to control topographic conditions and rainfall characteristics. Specific objectives were to test (i) the effects of a wide range of crust types and rain intensities on sloping runoff, considering antecedent soil moisture, and (ii) the biocrust resistance and soil loss through a splash erosive process of multiple crust types over a wide range of rain intensities. Based on previous work and 35 years of field observation, we hypothesize, as in Lopez-Canfin et al. (2022), that where biocrust development is not limited by soil instability or exposure to sun, the biocrust successional stages, from the earliest to the latest, could be as follow: incipient cyanobacterial, mature cyanobacterial, lichen biocrust dominated by *Squamarina lentigera* and/or *Diploschistes diacapsis*, and lichen biocrust characterized by *Lepraria isidiata*. Because biocrusts’ biomass, along with the soil’s organic carbon, aggregate stability and quantity of exopolysaccharides, increases along successional stages (Fischer et al., 2013; Colica et al., 2014), we hypothesized a larger biocrust-driven stabilization and lower erodibility in late-successional biocrusts, dominated by lichens or mosses, than in cyanobacteria-dominated early-successional stages and in physical crusts. We also hypothesized that, until the possible soil loss thresholds caused by biocrust break are reached, a positive linear relationship will exist between runoff and soil loss (measured as sediment content in runoff) with rainfall intensity (Lázaro et al., 2015). Although runoff will not always cause significant differences between biocrust types at high rain intensities (Rodríguez-Caballero et al., 2014), we expected that sediment content would show that late-successional biocrust has lower erodibility, as it has more organic matter and, in general, shows greater infiltration (Lázaro et al., 2021). We also expected that higher antecedent soil moisture would result in a higher runoff coefficient and higher soil loss rates (Chamizo et al., 2015). Nevertheless, because soil loss could disproportionately increase with regard to runoff if the biocrust breaks, we wondered whether an erodibility threshold associated with extreme rain intensities exists.

## 2. Materials and methods

We examined runoff and soil loss processes in laboratory, which was necessary to test a wide range of rain intensities crossed with a wide range of crust types while avoiding the topography effect. Carrying out the simulations in the laboratory limited the size of the sample units, since it was crucial for our purpose that the soil samples be undisturbed.

### 2.1. The field site

**Figure 1:**
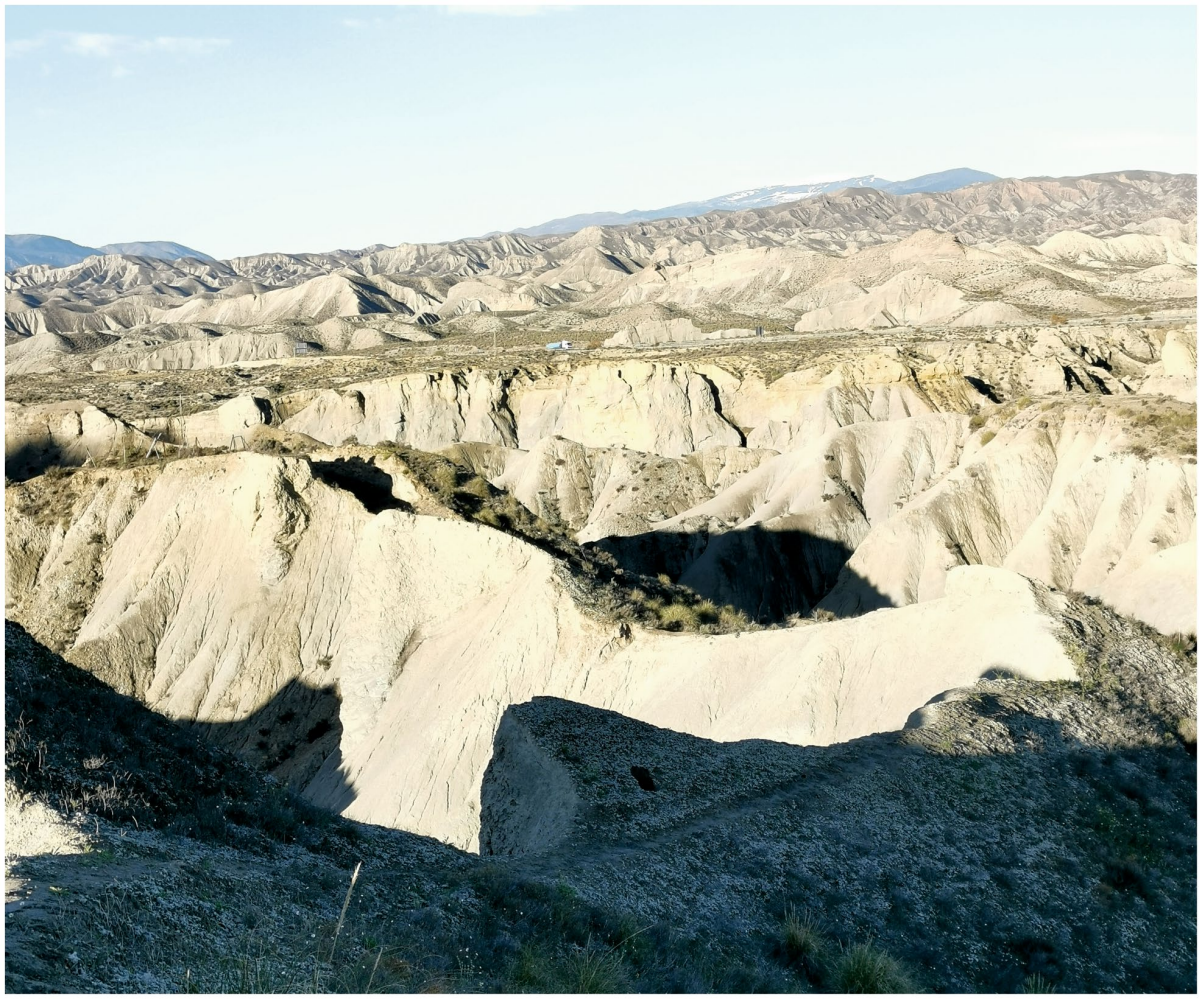
Characteristic landscape of the Tabernas Desert, Almería, Spain. The high mountains in the right of the background are part of the Sierra Nevada. Lower, in the foreground, it is possible to distinguish a lichenic biocrust.

The El Cautivo experimental area is a representative part of the Tabernas Desert (Almeria, Southeast Spain) with uneven topography due to a dense drainage net (Figure 1). This badlands area began developing in the late Pleistocene era (Alexander et al., 2008), and its main lithology is Tortonian marine marls (Cantón et al., 2001, 2002). The climate is semi-arid and thermo-Mediterranean. According to the official Tabernas meteorological station belonging to the ancient Spanish National Institute of Meteorology, located at 37° 03′ 10″ N, 2° 23′ 27″ W, 490 m a.s.l., the mean annual rainfall for the period 1967–1997 was 235 mm, with 36% interannual variability. Interannual variability for monthly rainfall reached up to 207%, and there are a large number of low-intensity events (Lázaro et al., 2001). These conditions favor biocrust development (Lázaro, 2004). Mean annual temperatures are 18° in Tabernas and 19° in our study area. The Walter-Lieth diagrams from the official Tabernas weather station (1967–1997) and our own station in the study site (1991–2004) were provided by Lázaro et al. (2008). Microclimatic weather stations started at El Cautivo in 2004 in representative microhabitats of different crust types, based on HOBO data loggers (Onset, United States). Each one includes an S-THB-M00x sensor (Onset, United States) for air temperature and relative humidity; an S-TMB-M0xx sensor (Onset, United States) for crust temperature; an S-SMA-M00x sensor (Onset, United States) for soil water content under the crust; an S-LIA-M00x sensor (Onset, United States) for photosynthetically active radiation; and a Rain-O-Matic-Pro tipping-bucket rain gauge with 0.25 mm resolution (Pronamic, Denmark) connected to a HOBO-Event data logger (Onset, United States). Some records for the period (2004–2021) in the station of cyanobacteria biocrust (the most open microhabitat) were: mean annual temperature 19.5°C; annual average of daily maximums 28.5°; annual average of daily minimums 12.3°; absolute maximum 49.3°; absolute minimum −5.8°; average of total annual rainfall 220 mm; total rainfall for the wettest year 401 mm, and total rainfall of the driest year 105 mm. General soil texture is 27% sand, 55% silt and 18% clay, showing moderate spatial variability (Cantón et al., 2003). Vascular vegetation is patchy, consisting of annual herbs dominated by *Stipa capensis* Thunb., drought-adapted dwarf shrubs, including endemism, such as *Helianthemum almeriense* Pau and *Hammada articulata* (Moq.) O. Bolòs and Vigo and grasses, mainly *Macrochloa tenacissima* (L.) Kunth (=*Stipa tenacissima* L), and it is only dominant in run-on sites. Within its patches, vegetation cover often oscillates between 25% and 50%, and its typical height is between 40 and 100 cm. Vascular vegetation extends over a third of the landscape. The south-to-west-facing hillslopes, with slope angles between 30° and 80°, are usually eroded and plantless, or supporting sparse individuals of *Salsola genistoides* Juss. ex Poir, accounting for another third. The rest is covered mainly by biocrusts (described below), which are also present in plant interspaces.

Erosion occurs in pulses, which vary greatly over time according rainfall magnitude and intensity, and in space (depending on landforms, vegetation and crust type). Cantón et al. (2003) indicated erosion rates of 6 g m^−2^ year^−1^ in the lower parts of the slopes with vegetation; 308 g m^−2^ year^−1^ in the eroded and nonvegetated slopes, and up to 26 g m^−2^ year^−1^ on the slopes covered with lichen-dominated biocrust (averages for 1994–1997).

### 2.2. Materials design

**Figure 2:**
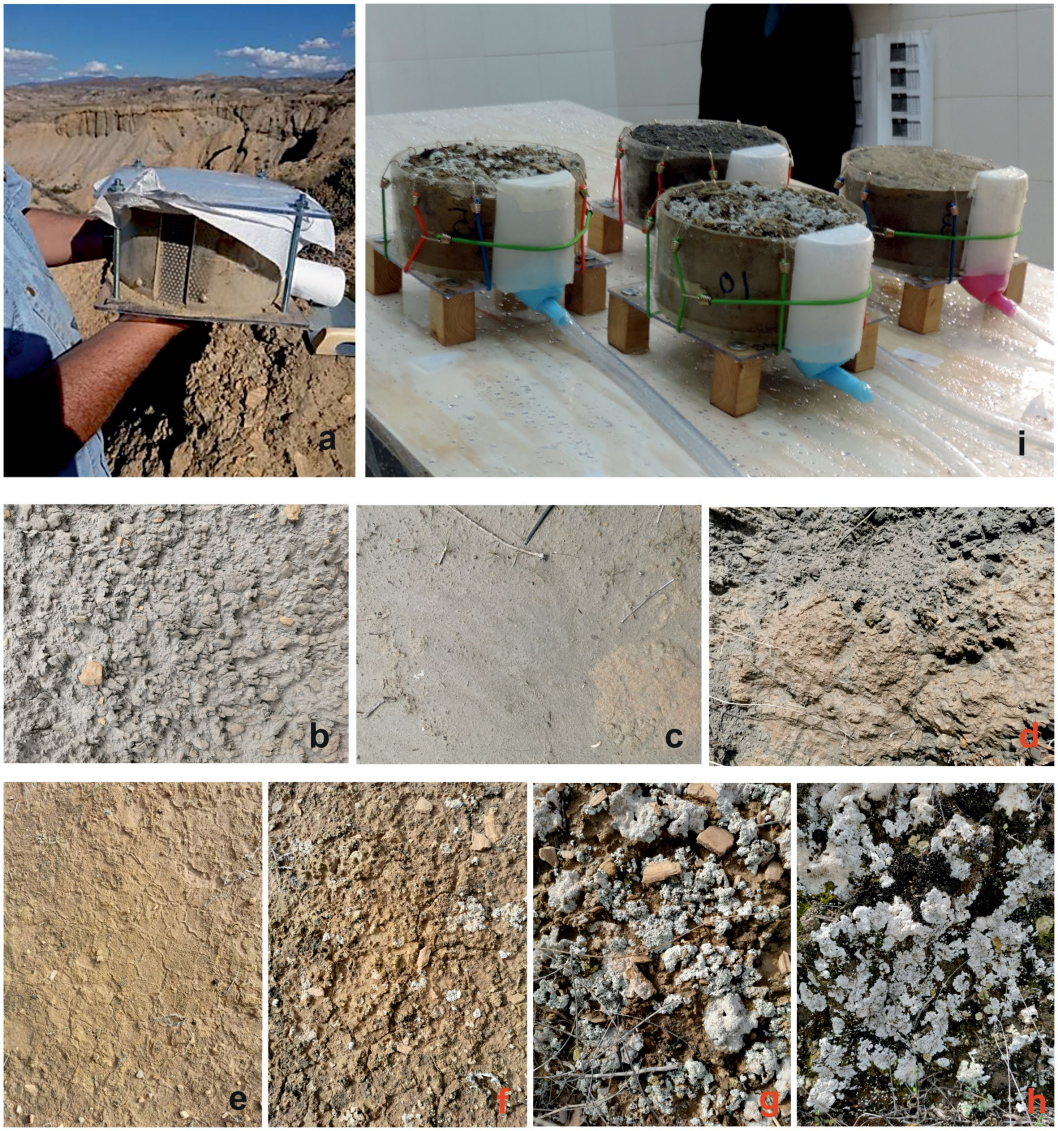
Undisturbed samples of crusted soil. **(A)** Sample immediately after being collected, conditioned for transport. Panels **(B–H)** correspond to the crust types: physical structural, physical depositional, physical island, incipient cyanobacterial, mature cyanobacterial, lichenic dominated by *Squamarina* and *Diploschistes*, and lichenic characterized by *Lepraria isidiata*, respectively. **(I)** Set of samples on the simulator table after an experiment, showing the collectors and pipes for runoff and sediments.

To take soil samples that maintain soil structure and surface microrelief, we built transparent methacrylate cylinders 20 cm in diameter and 10 cm in height, with a 3-cm opening along the entire height, covered with a stainless-steel curved plate with 2-mm holes spaced 3 mm apart to allow drainage and ensure soil retention (Figure 2A). The piece of methacrylate produced when cutting the opening was used afterwards to cover the steel plate in such a way that, in this case, the drainage came only from the uppermost 1 cm of soil. The cylinders had 5-mm walls and a sharp bottom edge at 45°, allowing us to insert the cylinders directly into the soil with a mallet. Once we inserted a cylinder into the soil, we horizontally inserted a stainless-steel plate made for this purpose with a handle that cuts the ground flush with the cylinder’s bottom edge, allowing us to remove it from the soil. Then, we covered the crust with several layers of soft paper before covering it with a methacrylate 25-cm-sided square plate with a hole in each corner. We turned the set, replaced the steel plate with another similar methacrylate plate, and fastened the two plates together with a screw and two nuts in every corner. These size of the sample units and procedures were successful for taking and transporting undisturbed crusted soil samples, allowing us to study the soil loss and the biocrust resistance under fully controlled conditions in the laboratory (we previously tried to collect undisturbed samples 30 cm in diameter, but the risk of cracking the surface during handling was too high). Despite the small size of the sample units the results were consistent with the current knowledge. On the other hand, this size was enough for a representative sample of the biocrust community (Eldridge and Greene, 1994).

### 2.3. Simulator setup

We aimed to produce at least three clearly different rain intensities in the range between the lowest producing runoff and the largest natural intensity (i.e., approximately between 10 and 300 mm/h). To achieve that, we preselected five nozzles of the HH-SQ model series from the extensive catalog of Spray System Co®, United States (a world leading nozzle manufacturer), because (i) they produce a fairly homogeneously distributed solid water cone with a relatively square basis and relatively abrupt borders; (ii) according to the manufacturer’s technical specifications, they produce rain intensities within the desired range; (iii) they produce drops between 0.1 and 5.0 mm, with 33% of the drops ranging from 0.8 to 1.4 mm and 30% ranging from 1.5 to 3.0 mm, clearly within the range of natural rainfall (Brandt, 1989); and (iv) they work with the flow of tap water, without any additional pump.

We tested the performance of those nozzles under various pressures of incoming water, as well as their water cones’ shape and size, using 13 collecting cups placed within 1 square meter on the simulator table, tilted 15°, during 10-min rainfalls. We accounted for the reduction of the cups’ area due to the table tilt. The nozzle was placed at a height of 5 m so that the drops fell almost vertically. A 0- to 6-bar manometer allowed us to control the inlet water pressure. Because they are quite contrasted and natural enough, we selected rain intensities of 18, 60, 120 and 240 mm/h, which were produced by the nozzle models and pressure of incoming water of 3.6SQ at 1 bar, 10SQ at 1 bar, 12SQ at 1 bar and 12SQ at 3 bar, respectively.

### 2.4. Sampling design and collection

We studied three physical and four biological crust types, distinguishable in the field with the naked eye, as listed below. The physical crusts are ordered from less to more stable—with less to more possibility of colonization. The biological crusts are ordered according to the hypothetical successional order.

- Structural physical crusts (Pe) from eroded hillslopes without vegetation or biocrusts, with hardly any soil. They are mainly regoliths resulting from rock weathering, with the surface sealed by raindrops, forming a very thin crust. They are mostly on the south-to-west-facing slopes (Figure 2B).
- Depositional physical crusts (Pd), which are a thicker physical crust formed from recurring episodes of deposition, compaction and sealing of successive silt layers. They are bare soil in depositional areas, mainly at the foot of eroded hillslopes (Figure 2C).
- Island physical crusts (Pi) from isolated sediment deposits (“islands”) surrounded by eroded regoliths. Deposition ceased because runoff circulates around the island, producing stabilization in a surface containing fines that could be suitable for colonization. Indeed, we observed biocrust in some of these “islands,” although we did not include biocrusts in these samples. This is the first study on Pi crust. The physical crusts are light gray, sometimes slightly beige in Pi (Figure 2D).
- Incipient cyanobacteria-dominated biocrust (I), having a smooth surface, a yellowish light brown color, and a biomass that is assumed to be low (Miralles et al., 2020). It occurs mainly in flat, sun-exposed and relatively trampled areas with very gentle slopes. It seems similar to Pd but with greater cohesion and mechanical resistance due to the biomass and organic matter (Figure 2E).
- Mature cyanobacterial biocrust (C), which is a brown cyanobacteria-dominated biocrust, with greater biomass and roughness than I and frequent but small pioneer lichens, such as *Endocarpon pusillum* Hedw., *Fulgensia desertorum* (Tomin) Poelt, *Fulgensia poeltii* Llimona and *Fulgensia fulgida* (Nyl.) Szat, are frequent, although the general lichen cover is low. Soil organic carbon (SOC) is higher than in I (14.1 g/Kg of SOC vs. 8.6 g/Kg in I), according to Miralles et al. (2020). It develops in any orientation, but it becomes dominant in sun-exposed, non-trampled places with gentle slope angles (Figure 2F).
- Lichen biocrust dominated by *Squamarina lentigera* (Web.) Poelt and *Diploschistes diacapsis* (Ach.) Lumbsch (S). Other lichens, such as *Buellia zoharyi* Galun and *Diploschistes ocellatus* Llimona, and *Psora decipiens* (Hedw.) Hoffm in the ecotone with MC, are frequent. It includes variable cyanobacterial cover. It is the most widespread lichen community and can form extensive patches, mainly in the upper half of north-to-east-facing hillslopes. It also occurs in the lower half of the hillslopes in the plant interspaces and even in slightly north-orientated pediments (Figure 2G).
- Lichen biocrust characterized by *Lepraria isidiata* (Llimona) Llimona and Crespo (L) and other lichens, such as *Squamarina cartilaginea* (With.) P. James, *Xanthoparmelia pokornyi* (Körb.) O. Blanco, A. Crespo, Elix, D. Hawksw. and Lumbsch and *Theloschistes lacunosus* (Rupr.) Sav. Dark microbial crust and mosses, such as *Didymodon luridus* Hornsch., *Grimmia pulvinata* (Hedw.) Sm. and *Tortula revolvens* (Schimp.) G. Roth, are also frequent. Several lichen species of the S biocrust are also common here. It occupies plant interspaces in the shaded north-facing slopes, often with relatively high slope angles (Figure 2H).

For every rain intensity except the highest, we collected a set of 28 unaltered crusted soil samples (four replicates × seven crust types) from typical field areas of every crust type. In all, we used 84 samples. The effect of the highest intensity was tested on all samples.

Once in the laboratory, we removed the layers of protective soft paper, anchored the base plate to the cylinder using elastic cords with hooks and sealed it to the cylinder with silicone. Then we labeled, air-dried and weighed the samples, clamping 3-cm-high wooden dowels at the four corners of the base for the runoff on the simulator table to circulate under the samples (Figure 2I).

### 2.5. Data generation

To disentangle the effect of rain intensity, as runoff and soil loss depend also on the quantity of water, we used rainfall durations of 80, 24, 12, and 6 min for the intensities of 18, 60, 120, and 240 mm/h, respectively, to ensure equal quantities of water in every experiment.

We simulated rainfall on four samples at a time, randomly selected from the 28 available, and we randomly placed them on the simulator table (Figure 2B). For each of these sets of four samples, we successively ran three experiments: first, we simulated on the dry samples. After waiting 20 min, we simulated again on the wet samples. After waiting another 20 min, we simulated a third time at an intensity of 240 mm/h. Table 1 shows the treatments.

**Table 1:** Samples and treatments.

Set	Crust	treat. 18d	treat. 18w	treat. 60d	treat. 60w	treat. 120d	treat. 120w	treat. 240w
Set 1	Pe	Pe18 1-4	Pe18 1-4					Pe18 1-4
Set 1	Pd	Pd18 1-4	Pd18 1-4					Pd18 1-4
Set 1	Pi	Pi18 1-4	Pi18 1-4					Pi18 1-4
Set 1	I	IC18 1-4	IC18 1-4					IC18 1-4
Set 1	C	MC18 1-4	MC18 1-4					MC18 1-4
Set 1	S	SD18 1-4	SD18 1-4					SD18 1-4
Set 1	L	Li18 1-4	Li18 1-4					Li18 1-4
Set 2	Pe			Pe60 1-4	Pe60 1-4			Pe60 1-4
Set 2	Pd			Pd60 1-4	Pd60 1-4			Pd60 1-4
Set 2	Pi			Pi60 1-4	Pi60 1-4			Pi60 1-4
Set 2	I			IC60 1-4	IC60 1-4			IC60 1-4
Set 2	C			MC60 1-4	MC60 1-4			MC60 1-4
Set 2	S			SD60 1-4	SD60 1-4			SD60 1-4
Set 2	L			Li60 1-4	Li60 1-4			Li60 1-4
Set 3	Pe					Pe120 1-4	Pe120 1-4	Pe120 1-4
Set 3	Pd					Pd120 1-4	Pd120 1-4	Pd120 1-4
Set 3	Pi					Pi120 1-4	Pi120 1-4	Pi120 1-4
Set 3	I					IC120 1-4	IC120 1-4	IC120 1-4
Set 3	C					MC120 1-4	MC120 1-4	MC120 1-4
Set 3	S					SD120 1-4	SD120 1-4	SD120 1-4
Set 3	L					Li120 1-4	Li120 1-4	Li120 1-4

Every set of undisturbed soil samples included 28 samples, with 4 replicates for each crust type. The dry samples of the first set were subjected to a simulated rainfall of 18 mm/h and then to a second equal rainfall on the wet sample (treatments “treat. 18d” and “treat. 18w,” respectively). The samples of sets 2 and 3 were used in the same way for 60 (dry and wet) and 120 mm/h treatments (dry and wet), respectively. All samples underwent an additional treatment, wet only, at 240 mm/h. All simulations used 24 mm of water and the same tilt (15°) for the samples. We used 84 samples in all; the treatment 240w used 12 replicates, and all other used 4 replicates.

We placed the samples on spaces previously marked on the simulator table within the rain cone, avoiding its center and edges. We maintained the table’s tilt at 15°. We coupled each sample to a purpose-made water and sediments collector. Transparent rubber tubes carried water and sediment from each sample to the edge of the table (Figure 2I). There, we recorded the time to runoff and then successive runoff volumes and times. We saved the runoff from each sample and experiment to quantify the sediments by filtering them under a vacuum using Buchner funnels and 0.45-micron filters (Millipore). We used the dry weight of sediments per liter of runoff as a measurement of soil loss and as an erodibility surrogate.

Once the rains had ended and the samples finished draining, we weighed the cylinders and labeled the difference between the moist and dry weights of every sample, expressed as a percentage of the dry weight, as “water content.” We used this value as a substitute for the sample’s porosity.

### 2.6. Data analyses

We analyzed runoff coefficient (RC, dimensionless), time to runoff (T_0_, seconds) and total soil loss (g/L) as dependent variables. We used Repeated-Measures Mixed Generalized Lineal Models (GLM) to test for differences in these dependent variables based on the fixed factors of crust type, rain intensity and antecedent soil moisture, as well as their interactions, including water content as a covariate approaching the soil porosity. First, we conducted two sets of GLM analyses: one distinguishing two general crust types, physical (Pe + Pd + Pi) and biological (I + C + S + L); and another set distinguishing the seven crust types (Pe, Pd, Pi, I, C, S, and L). The levels for the other factors were the same in both sets: 18, 60, and 120 mm/h for rain intensity and “dry” and “wet” for antecedent soil moisture. As simulations conducted on every sample were not independent, soil moisture was included as a within-subject factor, whereas crust type and rain intensity were considered between-subject factors. In these first two sets of GLMs, which included antecedent soil moisture as a factor, we only included the first three rain intensities because the experiments with 240 mm/h of intensity did not have their own set of samples and therefore could not be carried out on dry soil. Second, we conducted two additional sets of GLM analyses (distinguishing two and seven crust types) only in the experiments starting with wet soil, therefore not including the antecedent soil moisture factor. Since the simulations of 240 mm/h depended more on previous experiments than the other simulations starting on wet soil, 240 mm/h could not be considered a fourth level of intensity. Thus, we constructed two new variables. One was the presence or absence of the 240 mm/h run, and the other, the *Antecedent intensity*, which takes the same values as Intensity for the intensities under 240 mm/h and, for the records belonging to 240 take the value of the antecedent intensity used in that sample (i.e., 18, 60, or 120). The second set of GLMs considered crust type and antecedent intensity (instead of Intensity, to avoid including that of 240 mm/h as a fourth level) as between-subject factors, whereas the first new variable (the presence of a 240 mm/h run) was included as a within-subject factor. We considered differences to be significant when *p* < 0.05. We conducted the analyses using SPSS (IBM Company, United States).

We plotted the mean values of the dependent variables against the factors controlling them.

## 3. Results

### 3.1. Effects of a wide range of crust types and rain intensities on runoff

We verified that runoff generation was Hortonian: the transparency of the containers showed that when the runoff began, the lower limit of the wetted zone of the soil was more or less close to the surface and the soil under it remained dry.

Table 2 schematically shows the results of the GLM analyses. The GLMs based on different data sets (i.e., distinguishing only between physical and biological crusts or distinguishing the seven crust types) showed quite a few coincidences but also some differences.

**Table 2:** Synthetic results from the 12 GLM analyses, showing the significant (>95%) effects in every case.

Data	Crust categories	Dependent variable	Effect	Statistic and significance	
				F.	*p*
Exclud. int. 240	2	Runoff coefficient	Intercept	8.607	0.000
			Intensity	3.531	0.032
			Crust (2)	7.553	0.007
			Antecedent moisture	73.285	0.000
Exclud. int. 240	2	Soil loss	Intercept	5.728	0.000
			Crust (2)	53.366	0.000
Exclud. int. 240	2	Time to runoff	Intercept	56.701	0.000
			Intensity	239.105	0.000
			Antecedent moisture	130.780	0.000
Exclud. int. 240	7	Runoff coefficient	Intercept	5.062	0.000
			Water content	8.725	0.004
			Intensity	8.200	0.001
			Crust (7)	5.161	0.000
			Antecedent moisture	104.623	0.000
			Intensity*Crust (7)	3.485	0.000
Exclud. int. 240	7	Soil loss	Intercept	16.740	0.000
			Crust (7)	47.873	0.000
			Intensity	7.180	0.001
			Antecedent moisture	9.719	0.002
			Intensity*AntMoisture	10.349	0.000
			Intensity*Crust(7)	13.844	0.000
			Crust(7)*AntMoisture	8.844	0.000
			Inten.*Crust (7)*Anteced.Moist.	10.382	0.000
Exclud. int. 240	7	Time to runoff	Intercept	5.440	0.000
			Intensity	7.211	0.001
			Antecedent moisture	155.213	0.000
Excluding dry	2	Runoff coefficient	Intercept	4.105	0.000
			Antecedent intensity	6.557	0.002
			Crust (2)*Ante.Intensity	3.330	0.039
			Presence of 240 run	8.779	0.004
Excluding dry	2	Soil loss	Intercept	10.009	0.000
			Antecedent intensity	3.869	0.023
			Crust (2)	57.611	0.000
			Presence of 240 run	8.752	0.004
Excluding dry	2	Time to runoff	Intercept	43.493	0.000
			Antecedent intensity	83.954	0.000
			Ant.Intensity*Presence 240	91.966	0.000
			Presence of 240 run	233.371	0.000
Excluding dry	7	Runoff coefficient	Intercept	2.385	0.000
			Antecedent intensity	9.800	0.000
			Presence of 240 run	14.829	0.000
Excluding dry	7	Soil loss	Intercept	8.146	0.000
			Antecedent intensity	12.810	0.000
			Crust (7)	19.645	0.000
			Presence of 240 run	26.222	0.000
			Crust(7)*Ant.Intensity	2.088	0.022
			Water content	9.583	0.002
Excluding dry	7	Time to runoff	Intercept	17.852	0.000
			Antecedent intensity	97.503	0.000
			Presence of 240 run	296.036	0.000
			Ant.Intensity*Presence 240	107.565	0.000

The runoff coefficient (dimensionless), soil loss (g/L) and time to runoff (s) were successively used as dependent variables. In the first two groups of GLM (distinguishing two and seven crust types, respectively), the factor *antecedent soil moisture* was included, whereas the runs with 240 mm/h intensity were not included. In the other two GLM groups (also distinguishing two and seven crust types), the 240 mm/h runs were included, but antecedent moisture was not.

**Figure 3:**
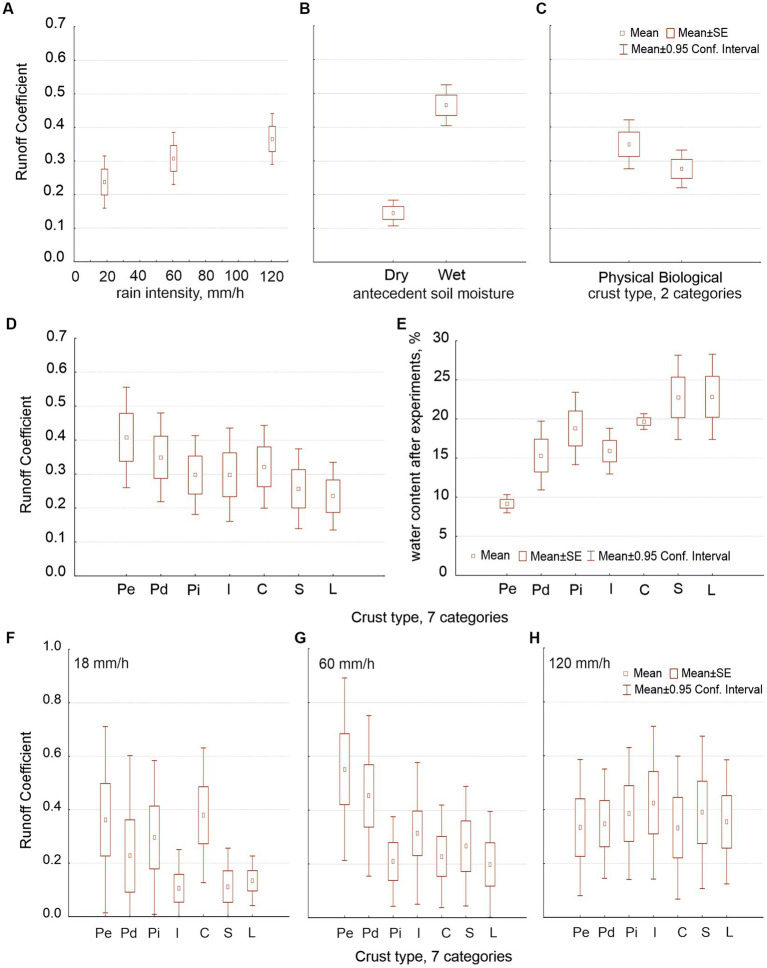
Average values of runoff coefficient (RC) as related to rain intensity (distinguishing three intensities: 18, 60 and 120 mm/h; graph **A**), with antecedent soil moisture (dry or wet; graph **B**), and with crust type, with two (graph **C**) and seven types (graph **D**). Graph **(E)** shows the water content after experiments in relation to the seven crust types. Graphs **(F–H)** show the interaction of the effects of intensity and crust type on RC.

Runoff coefficient (RC) increased with rain intensity and with soil moisture (Figures 3A,B) and decreased along the hypothetic crust succession: RC was lower in biocrusts than in physical crusts and in lichenic biocrusts than in cyanobacterial ones (Figures 3C,D). Differences between both data sets were as follows: (i) The water content (substitute for soil porosity) had a significant effect on RC only when we distinguished seven crust types (because it varied significantly when compared with crust type; Figure 3E). (ii) The intensity affected how much crust type affected RC (Figures 3F–H show this interaction).

**Figure 4:**
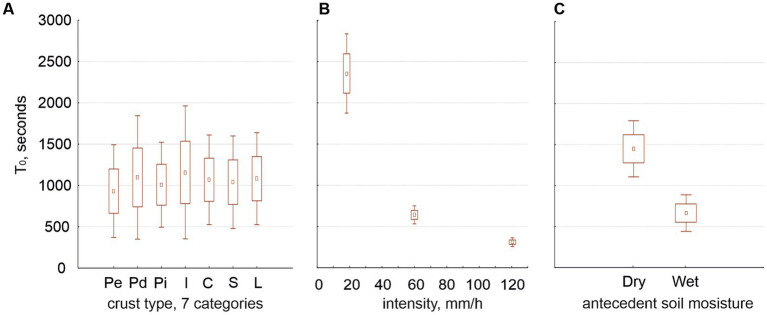
Average of the times to runoff (T_0_, seconds) in relation to the seven crust types (graph **A**), the three rain intensities (graph **B**) and the two antecedent soil moistures (graph **C**).

Regardless of whether we consider two or seven crust types, the time to runoff (T_0_) was independent from the crust type (Figure 4A) but highly dependent on intensity (Figure 4B) and antecedent soil moisture (Figure 4C). No other factor affected T_0_ whether we considered two crust types or seven.

**Figure 5:**
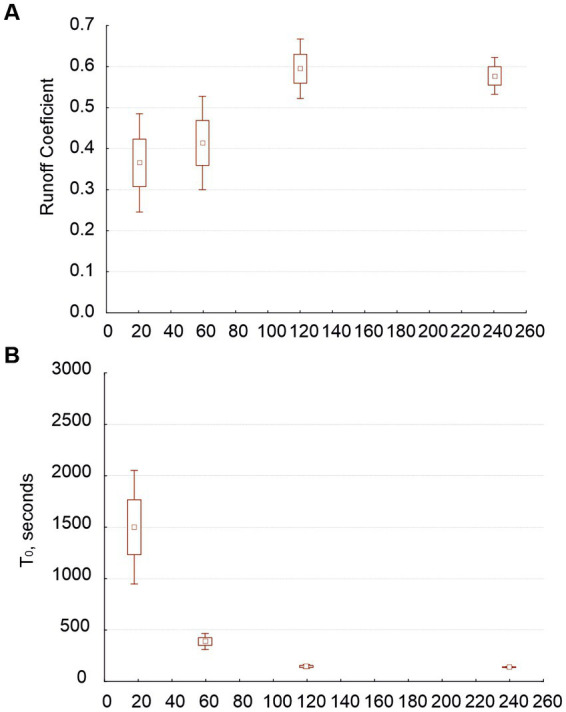
Average values for runoff coefficient (graph **A**) and time to runoff (graph **B**) as related to rain intensity when we plotted four rain intensities (18, 60, 120, and 240 mm/h; only experiments starting on wet soil).

When we analyzed the data sets including the experiments with 240-mm/h rain intensity but not those beginning with dry soil, the patterns of the dependent variables vs. crust types were similar to those we found in our analyses of the first sets. Nevertheless, RC was 0.2 (or more) greater (as we conducted these experiments on wet soil), and T_0_ was half or a third of the times found in the first sets. RC depended on intensity and on the interaction between intensity and crust type in distinguishing the two types of crust, but in distinguishing seven, RC was independent from crust type and only marginally dependent on its interaction with intensity. Note that RC increased with intensity only up to 120 mm/h (Figure 5A). At high intensities (120 and 240 mm/h), we found very similar RC for both physical and biological crusts. T_0_ was strongly affected by intensity (Figure 5B); however, the T_0_ at 120 mm/h was very close to the minimum reached at 240 mm/h. The way in which the largest intensity affected T_0_ was modulated by the antecedent intensities used on the same samples.

### 3.2. Biocrust resistance and soil loss in multiple crust types over a wide range of rain intensities

**Figure 6:**
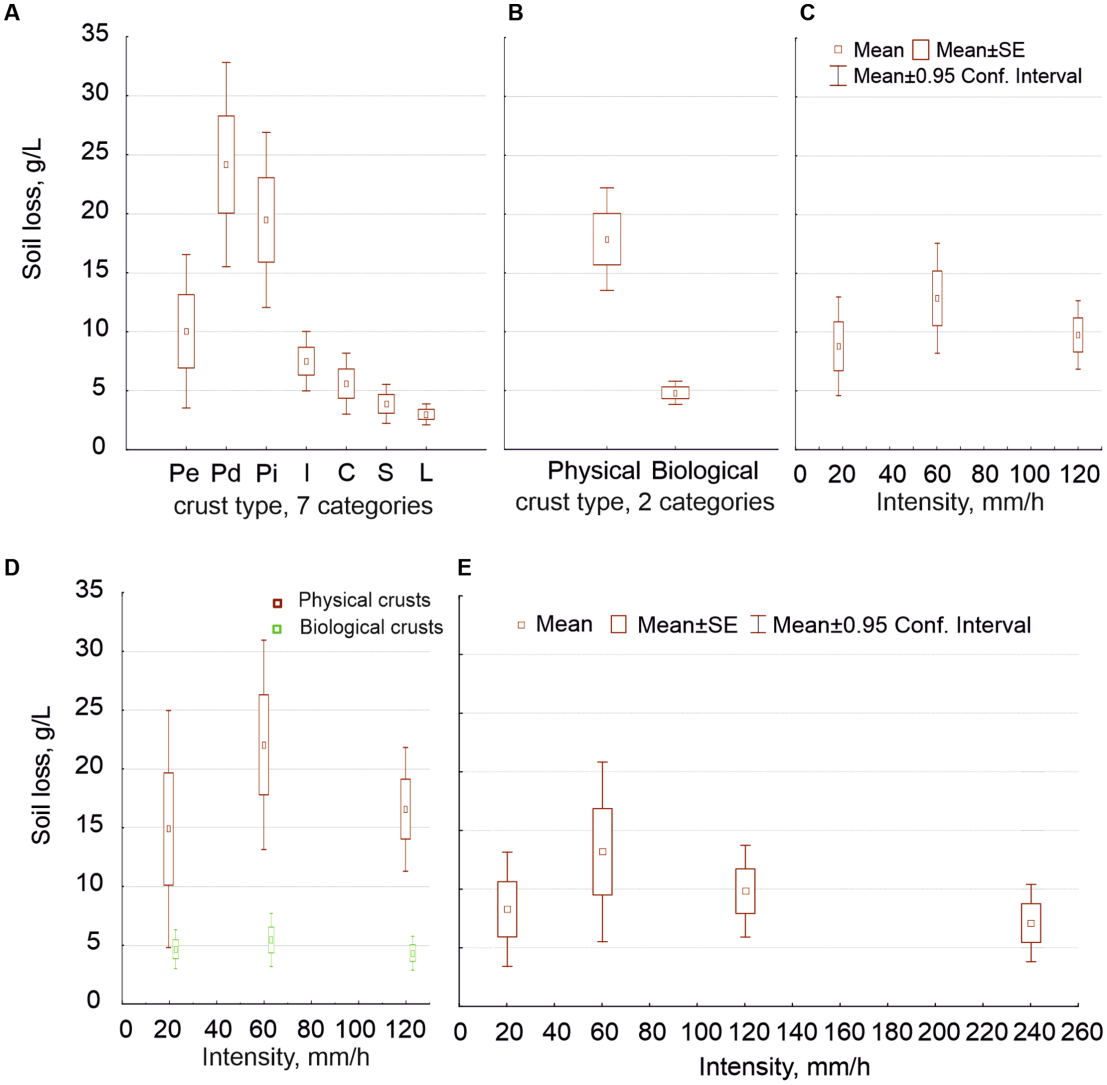
Average of soil loss values (g/L) in relation to the seven crust types (graph **A**) and the two general crust types (graph **B**); as well as to the three rain intensities, with all of the data together (graph **C**) and separating the physical from the biological crusts (**D**); and to the four rain intensities (graph **E**).

With regard to soil loss, for the first data sets (including antecedent soil moisture but not the 240 mm/h intensity), the crust type’s effect was highly significant independently of the number of crust types considered (Figures 6A,B). The difference in soil loss was one order of magnitude between the most erodible physical crust and the least erodible biocrust. No other factor had an effect on soil loss when we distinguished two crust types, not even intensity (soil loss at 60 mm/h was greater but not significantly). However, when we distinguished the seven crusts, the three factors (crust type, rain intensity and antecedent moisture) were significant as well as all the second-order interactions between factors and even the third order interaction (Table 2). Interestingly, the maximum soil loss occurred at 60 mm/h, decreasing at greater intensities (Figure 6C). This variation in soil loss was due to the physical crusts; the biological ones showed high protection against soil loss at all intensities; the Figure 6D shows the interaction between intensity and crust type.

When we included the 240 mm/h intensity (but only the wet experiments), soil loss depended again on crust type but was also significantly affected by intensity (Figure 6E), so in the case of two crust types as in the case of seven, decreasing past 60 mm/h, as above. Moreover, distinguishing seven crust types, the interaction between intensity and crust type resulted significant, and also the soil porosity, approached by the water content. Soil loss was slightly lower at 240 mm/h than at 120 mm/h and similar to that at 18 mm/h (Figure 6E). This pattern in the relationship between soil loss and intensity occurred due to the physical crusts, as we showed in the first sets. No biocrust was broken, not even at the intensity of 240 mm/h.

## 4. Discussion

Maintaining the rainfall volume and the slope angle, we simulated a series of rainfall intensities on seven types of crusted, undisturbed soil samples in the laboratory. In our study area, differences in runoff (Lázaro and Mora, 2014; Lázaro et al., 2015) and soil loss based on biocrust types and rain intensity have been determined from *in situ* field rainfall simulations respectively and in field plots under natural rainfall (Chamizo et al., 2012b; Lázaro et al., 2021). However, the numbers of considered crust types and rain intensities were much fewer than those used here. Moreover, this is the first study of the late-successional Lepraria community using simulated rainfall. Despite the small size of the sample units, none of the results contradict prior knowledge, which is interesting by itself and gives plausibility to the new results.

### 4.1. Effects of crust type and rain intensity on runoff

Our experiments confirmed previous knowledge: runoff coefficient (RC) increased with rain intensity and soil moisture (Figures 3A,B) and decreased along the hypothetical crust succession (Figures 3C,D). Water content (soil porosity) increased along the hypothetical crust succession (Figure 3E). Time to runoff (T_0_) was independent from the crust type (Figure 4A) with some exceptions, but highly dependent on intensity (Figure 4B), antecedent soil moisture (Figure 4C), and their interaction. Our experiments originated new findings: intensity interacted with crust type (which is consistent with the findings of Guan and Cao, 2019) but in such a way that beyond 120 mm/h the crust was unable to affect the RC (Figures 3F–H). T_0_ decreased with intensity only up to 120 mm/h (Figures 5A,B) as well.

It is known that runoff increases with rain intensity, and an increment of RC could be also expected (Chaplot and Le Bissonnais, 2003; Martínez-Murillo et al., 2013). However, we found that RC did not increase beyond 120 mm/h. Note that similar RC values for 120 and 240 mm/h (Figure 5A) mean that the runoff increased because the rain did. According to Defersha and Melesse (2012), although runoff rate increases with rainfall intensity, RC does not. Lázaro et al. (2015), through *in situ* rain simulations at our study area on plots from 1 to 4 m long, found that RC does not increase with rain class (involving volume and intensity), although runoff does. They proposed that infiltration increases with intensity and that there would be a threshold of intensity’s effect on RC at around 77 mm/h (the greatest intensity they used). Here, the difference between RC at 60 and 120 mm/h was only marginally significant (Figure 3A) when we considered three intensities, but when we plotted four, RC at 120 mm/h was significantly higher than at 60 mm/h and equal to that at 240 mm/h (Figure 5A), suggesting that the threshold could be over 77 mm/h and close to 120 mm/h.

Despite the high intensities, we still found lower RC in biocrusts than in physical crusts (Eldridge and Greene, 1994; Solé-Benet et al., 1997; Belnap et al., 2013), which is due to three main nonexclusive mechanisms: (i) the increase in surface roughness (Kidron et al., 2012; Rodríguez-Caballero et al., 2012); (ii) the increase in large, irregular, elongated and interconnected pores (Miralles-Mellado et al., 2011); and (iii) the increase in soil organic carbon (Gao et al., 2019). However, in sandy soils, biocrusts increase RC in comparison to physical crusts, as they accumulate finer soil particles and have a greater capacity for both pore clogging and water holding (Kidron, 2007; Malam Issa et al., 2009, 2011; Wu et al., 2012).

Unlike in nature, we found higher RC in Pd than in Pe crust because, in our experiments, both had the same slope and the Pe has a greater roughness. In general, cyanobacterial crusts (I and C) had a lower RC than physical crusts because their roughness is greater (Chamizo et al., 2010). Lichenic crusts (SD and Li) had an even lower RC due to their greater organic matter, greater roughness (Rodríguez-Caballero et al., 2012) and greater capacity to swell upon wetting, causing pore clogging (Kidron et al., 1999). The intensity threshold, close to 120 mm/h, that we found beyond which the crust’s effect on runoff becomes irrelevant (because rainfall greatly exceeds the infiltration capacity) agrees with what Rodríguez-Caballero et al. (2013) found at a large-plot scale.

According to Keya and Karim (2020), RC increases with the clay content in laboratory simulations with disturbed soil samples. However, here, RC decreased along the crust succession despite the fact that fines tended to increase (Miralles-Mellado et al., 2011). This agrees with the results from large runoff plots under natural rainfall (Lázaro et al., 2021) and probably occurs because porosity (Miralles-Mellado et al., 2011) and surface roughness (Chamizo et al., 2010) also increase along the biocrust succession and both favor infiltration. This opposite result to that of Keya and Karim (2020) shows the great difference of simulating on undisturbed soil samples.

We find that antecedent soil moisture had a positive effect on runoff and RC on any surface (Chamizo et al., 2013; Lázaro et al., 2015) and a negative effect on T_0_ (Mayor et al., 2009). T_0_ was higher in biocrust than in physical crusts (agreeing with Chamizo et al., 2010) and potentially decreased with increasing intensity, especially when we started with dry soil. Calvo-Cases et al. (2005) observed T_0_ of 30 and 10 min at 25 and 40 mm/h, respectively. An estimation from Figure 4B shows comparable values of T_0_ from our experiments: about 36 min at 25 mm/h and 17 min at 40 mm/h. Since T_0_ potentially decreases with increasing intensity (Figures 4B, 5B), one would expect the decrease in T_0_ to become nonsignificant beyond a certain intensity, as we found here at 120 mm/h; however, we have not found that threshold in the literature.

### 4.2. Biocrust resistance and soil loss

The soil loss strongly decreased along the hypothetical crust succession, up one order of magnitude (Figures 6A,B), as expected (Lázaro et al., 2008; Lázaro and Mora, 2014). It also increased with intensity up to 60 mm/h, decreasing afterward (Figure 6C): a decoupling between runoff and soil loss occurred at high intensities, mainly in physical crusts. Biocrusts showed a similar pattern, but their soil loss was much lower and practically independent from rain intensity, as differences were not significant. This was a new or little-known result: biocrust provided high protection at all intensities (Figure 6D), even at 240 mm/h (Figure 6E).

It is well known that biocrusts reduce soil loss (Eldridge and Leys, 2003, among many others). This is so important that it has consequences at the landscape scale (Lázaro et al., 2022). Eldridge and Kinnell (1997) empirically found that splash erosion potentially decreased when biocrust cover increased. A 20% cover can be enough to decrease erosion significantly (Calvo-Cases et al., 1991). Biocrust increases soil stability through (i) excreted exopolymers and anchorage structures (Warren, 2003); (ii) the increase in roughness and infiltration, reducing runoff speed (Kidron et al., 1999), thereby reducing the soil loss caused by runoff; and (iii) absorption of the kinetic energy of raindrop impacts (Li et al., 2005). That soil loss increases with rain intensity is also known (Lázaro and Mora, 2014; Keya and Karim, 2020). We had guessed an intensity threshold beyond which the soil loss could be disproportionately large with respect to the runoff due to the crust breaking. However, although runoff increased with intensity (agreeing with Chamizo et al., 2012b; Rodríguez-Caballero et al., 2013), soil loss only increased up to 60 mm/h in any crust type, decoupling after that from runoff and decreasing mainly in physical crusts; moreover, no biocrust was macroscopically broken at any intensity. These were novel results, although Defersha and Melesse (2012) found, in agricultural soil samples that did not retain their natural structure, that soil loss does not show any trend with rainfall intensity except in a soil type, in which the soil loss increased up to 70 mm/h and then decreased for 120 mm/h. The existence of that maximum of soil loss could be a general phenomenon, but its exact location in the range of rain intensity is probably related to the infiltration capacity of the soil.

As for the physical crusts, we found greater erodibility in Pd than in Pe (Figure 5A), despite the fact that Pe undergoes much higher erosion in field (Lázaro et al., 2008). This is because, in nature, Pd has much lower slope and is in aggradation most of the time. Pi, unlike the other types of physical crusts, increased its soil loss as intensity increased, possibly due to its lower density (higher values of water content, Figure 3E). This is the first study including Pi, which is similar to but thinner than Pd, since no deposition occurs with runoff flowing around these isolated sediment patches. Field observation shows that biocrusts do not develop in places undergoing erosion, nor in those in aggradation; they need stability for a sufficient time (Lázaro et al., 2022). However, it is not rare to find some biocrusts in these “islands,” although we avoided them during sampling.

According to the review of Martínez-Murillo et al. (2013), the average sediment concentration in runoff, based on field rain simulations, is 17.8 g/L at the Tabernas badlands. The average soil loss we found in our physical crusts is similar (Figure 6B).

Due to the size of our sample units, runoff did not reach a sufficient flow and speed to exert a significant erosive force. Soil loss is due to rain splash, which detaches soil particles (Kinnell, 2005). Although greater intensity implies more raindrops, more detached particles could give rise to a particle layer that armors the surface from further particle detachment. Moreover, for high rain intensities, a film of water would develop on the surface, protecting it against the impact of raindrops, as the infiltration excess is greater when rain intensity increases. Poesen and Savat (1981) attributed decay of raindrop detachment to a water film of undrained water due to the rain’s duration.

We wondered whether this protective ponding could occur because the limited width of the sample containers’ drain outlets could retain the runoff from a certain flow rate. However, the average number of 2-mm holes in the steel plate was 7.5, representing an exit of 1.5 cm in width for a sample 19 cm in diameter, a ratio of 0.079 (7.89%), which is considerably larger than those used in semi-arid Spain to date. Including bounded and open-runoff plots, for both simulated and natural rainfall, the used ratios were 0.056 (Calvo-Cases et al., 1991; Solé-Benet et al., 1997; Chamizo et al., 2012b), 0.04 (Chamizo et al., 2012b; Rodríguez-Caballero et al., 2013; Chamizo et al., 2017), 0.05 (Lázaro and Mora, 2014; Lázaro et al., 2015) and 0.02 (Rodríguez-Caballero et al., 2014; Cantón et al., 2020). Similarly, the approximate ratio of the multiple-sized runoff plots used in Negev, Israel, ranged between 0.015 and 0.05 (see, among others, Kidron and Yair, 1997; Kidron et al., 2003, 2012; Kidron and Büdel, 2014). Our preliminary assays showed that the possible obstruction of holes in the steel plate during an experiment was infrequent. Besides, even in the case where half of the holes were blocked during an entire event, our drain capacity would be within the range of the usual runoff collectors. On the other hand, in surface concavities, where water accumulates, new lines of holes come into play, locally doubling or tripling the drainage ratio. Thus, the protective water film is probably a real phenomenon at high intensity.

The small size of our sample units presented several advantages: (i) While enabling sufficiently representative samples of the biocrust communities (Eldridge and Greene, 1994), it allowed us to keep their soil structure and crust integrity, thus enabling laboratory experiments relatively equivalent to *in situ* rain simulations, but with full control of factors such as the slope or wind, and greatly simplifying logistics. (ii) It can allow researchers to obtain runoff and soil loss data from lower intensities than in the case of larger plots typical in field. (iii) Mass movements, rills, gullies or piping cannot form. Therefore, any soil loss occurred due to splash and diffuse runoff, which are the erosive processes most dependent on surface type, as well as the most widespread (Regües et al., 1995).

### 4.3. Biocrust types as successional stages and microbiology

In the same area and four biocrust types or successional stages considered here, Miralles et al. (2020) found the following proportions of the seven predominant bacterial phyla: Actinobacteria showed 14.4%, 9.8%, 12.7%, and 10.9% for the biocrusts I, C, S, and L, respectively, being the only phylum with a fuzzy relationship. Two other phyla tended to increase: Proteobacteria (14.8, 13.2, 16.6, and 17.1, respectively) and Bacteroidetes (14.6, 14.3, 18.3, and 16.1, respectively). Two others increased consistently: Planctomycetes (6.0, 5.9, 8.2, and 14.4) and Acidobacteria (5.7, 7.0, 10.4, and 11.6). On the other hand, two others decreased consistently along the hypothetical succession: Cyanobacteria (12.4, 21.9, 6.4, and 2.2) and Chloroflexi (11.3, 9.7, 8.8, and 6.4, respectively). Cyanobacteria, which reaches the highest proportion, strongly decreases with lichen development. The differences in bacterial communities among biocrust types were significant. These four biocrust types were the main factors differentiating the biomolecules they excrete (Miralles et al., 2017), and these metabolites would influence the variation of the microbiota over time (Boustie and Grube, 2005).

Soil microfungi from our study area, under three lichens (*Psora decipiens*, *Squamarina lentigera*, and *Diploschistes diacapsis*), cyanobacterial biocrust, and non-biocrusted soil, at two habitats (sun-exposed and shadier), included Zygomycota (6 species), sexual Ascomycota (11), asexual Ascomycota (59), and Basidiomycota (1); in all, 77 species of 46 genera, plus five unidentified (Grishkan et al., 2019). Lichens hosted a significantly greater density of isolates than cyanobacteria, particularly *Diploschistes*. Melanized fungi with large multicellular spores, although dominant, were less abundant than in the Negev Desert (Israel) and the Tengger Desert (China), while the thermotolerant *Aspergillus* spp. were relatively abundant. At each habitat, the contribution of aspergilli was significantly higher on the bare surfaces, the lowest occurring in the Cyanobacteria and *Diploschistes* at the shadier position, where the typical melanized species peaked. When we sampled soil profiles from 0 to 30 cm depth (Grishkan et al., 2020), we found 116 species from 60 genera of microfungi, or 142 species from 68 genera (Grishkan et al., 2021). While melanized species dominated the uppermost communities, *Aspergillus* spp. was mainly at 1–5 cm, and mesophilic *Penicillium* spp. at 10–20 cm, peaking in the shadier profiles. The effect of soil depth on microfungi was highly significant.

If our crust types can be assumed as successional stages, our results configure a series of processes over time: an increase in infiltration, soil porosity, and water holding capacity, and a decrease in soil loss, which is associated with a decrease in the loss of nutrients (Lázaro and Mora, 2014). These processes, consistent with the literature, imply an increment of water availability, aeration, stability, and retention of fine soil particles and nutrients, and such changes through crust succession are very probably part of the explanation of the time evolution of the microbiota. Moreover, Grishkan et al. (2020, 2021) discuss the influence of infiltration on the vertical distribution of microfungi taxa.

## 5. Conclusion

By collecting multiple undisturbed soil samples of 20 cm in diameter from three physical and four biological crust types and using laboratory rainfall simulation, we studied the erodibility, due mainly to splash, of every crust across a large range of rain intensities: 18, 60, 120, and 240 mm/h, keeping the slope constant. Despite the small size of the sample units, the results were consistent with previous research at less spatial resolution, as the positive effect of rain intensity and antecedent soil moisture on RC; or the fact that biocrust decreased RC, except in the case of extraordinarily intense rains, this effect being stronger in the most advanced successional stages; or the strong effect of biocrusts protecting the soil.

This provides plausibility to the novel findings. A threshold of rain intensity beyond which RC remained stable was established. The reduction of soil loss provided by biocrusts, which was larger with more advanced succession, remained almost constant regardless of rain intensity and antecedent soil moisture. At high rain intensity, runoff decoupled from soil loss and there was a threshold of soil loss in relation to rain intensity due to the protective effect of a film of water and detached soil particles on the surface. Its location in the intensity range would be related to the soil infiltration capacity. The protective film’s thickness and effectiveness are presumably dependent on the slope. Thus, an adequate description of such a threshold requires further study using different slope angles. Our results provided empirical evidence on the crucial role of biocrusts in reducing soil erosion, which is particularly important in a climate-change context where more extreme events are predicted.

This research highlights the consistency and usefulness of considering biocrust types as successional stages when studying interrelationships or processes involving biocrusts and their habitats. Biocrust succession might not occur in the most limited habitats (or it might take longer than human life), where colonizing or intermediate biocrust communities can be virtually permanent. Despite this, biocrust succession is still the better conceptual model to explain a lot of biological, microbiological, physical and chemical changes occurring over time in soil and on the soil surface.

## Data availability statement

The raw data supporting the conclusions of this article will be made available by the authors, without undue reservation.

## Author contributions

RL conceived of the idea and designed the methods. CG, CR, and RL calibrated the simulator, elaborated the data, conducted analyses, and created the graphs. CG and RL took the samples in the field, conducted the experiments in the laboratory, and wrote a preliminary version of the text. RL wrote the final version, which CG and CR reviewed. All authors contributed to the article and approved the submitted version.

## Funding

The work was supported by the research projects DINCOS (CGL2016-78075-P) and INTEGRATYON, sub-project 2 (PID2020-117825GB-C22), both funded by the Spanish State Program for Scientific Research; by the European Regional Development Fund; and by the BAGAMET project (Andalusian Plan for Research, Development and Innovation—PAIDI—2020. Junta de Andalucia; call for projects 2020. File number: P20_00016). Consuelo Rubio’s participation was possible thanks to doctoral student contract FPU18/00035. The funding sources were not involved in the study design; in the collection, analysis and interpretation of data; or in the writing of the report.

## Conflict of interest

CG was employed by SGS Tecnos SAU.

The remaining authors declare that the research was conducted in the absence of any commercial or financial relationships that could be construed as a potential conflict of interest.

## Publisher’s note

All claims expressed in this article are solely those of the authors and do not necessarily represent those of their affiliated organizations, or those of the publisher, the editors and the reviewers. Any product that may be evaluated in this article, or claim that may be made by its manufacturer, is not guaranteed or endorsed by the publisher.
